# Para Onde Vamos com os Produtos Naturais? Explorando o Verdadeiro Potencial de Novos Medicamentos Derivados de Plantas no Campo Cardiovascular

**DOI:** 10.36660/abc.20220430

**Published:** 2022-07-29

**Authors:** Artur Santos-Miranda

**Affiliations:** 1 Universidade Federal de Minas Gerais Belo Horizonte MG Brasil Universidade Federal de Minas Gerais, Belo Horizonte, MG – Brasil

**Keywords:** Doenças Cardiovasculares/fisiopatologia, Antiarrítmicos, Fatores de Risco, Cardiotoxicidade, Plantas Medicinais, Técnicas de Patch-Clamp, Terpeno, Carvone

As doenças cardiovasculares são uma das principais causas de morte em todo o mundo. Na população brasileira, estima-se que aproximadamente 41,6% das mulheres e 63,5% dos homens tenham risco médio a alto de desenvolver doenças cardiovasculares nos próximos 10 anos.^1^ As arritmias cardíacas são manifestações comuns das doenças cardiovasculares e configuram importante causa de morbidade e mortalidade entre as cardiopatias. Após a classificação de Vaughan-Williams dos medicamentos antiarrítmicos com base em suas ações farmacológicas, várias novas terapias e drogas foram propostas, visando alcançar uma alta eficácia com o mínimo de efeitos adversos. No entanto, os tratamentos com drogas antiarrítmicas e outros agentes usados para tratar doenças cardiovasculares, como insuficiência cardíaca, são frequentemente propensos a respostas adversas pró-arrítmicas.^2,3^ Além disso, complicações cardíacas, como arritmias, também são observadas no tratamento de outras patologias, como o câncer, e também durante o uso de antidepressivos.^4,5^

Os medicamentos derivados de plantas são há muito tempo utilizados na medicina tradicional/alternativa para os mais diversos fins. Seus usos se correlacionam com vários fatores, incluindo tradição familiar, idade, sexo, educação, status socioeconômico e o fracasso das terapias convencionais.^6^ Entre os medicamentos à base de plantas, diferentes tipos de terpenos têm sido explorados como fragrâncias/repelentes, mas também de acordo com seu potencial médico no tratamento de doenças parasitárias, infecções bacterianas, cicatrização de feridas e como agentes antioxidantes e anti-inflamatórios.^7^ Além disso, as propriedades antiarrítmicas de alguns terpenos foram abordadas usando modelos in vitro e experimentais^8,9^ enquanto outros terpenos podem realmente ter atividade pró-arritmogênica.^10^

Nesta edição dos Arquivos Brasileiros de Cardiologia, as propriedades antiarrítmicas do monoterpeno (-)-Carvona foram exploradas *in vitro* e *ex vivo* usando diversas preparações que vão desde ensaios celulares até o órgão isolado.^11^ A (-)-Carvona evocou um efeito inotrópico negativo nos átrios de forma dependente da concentração e reduziu a contratilidade de corações isolados após exposição aguda ao terpeno. O perfil do eletrocardiograma (ECG) de corações isolados expostos a essa droga foi marcado, com diminuição da frequência cardíaca, aumento do intervalo PR e QTc. Em preparações de cardiomiócitos isolados, a (-)-Carvona levou a uma diminuição da corrente de cálcio do tipo L, do transiente de cálcio intracelular e contração celular, o que se alinha bem com os achados no coração e nos átrios isolados. Além de seus achados, a (-)-Carvona reduziu a gravidade das arritmias em um modelo experimental de corações isolados expostos a um meio com alto teor de Ca2+. Os autores concluíram que a (-)-Carvona tem uma atividade antiarrítmica promissora ao diminuir a entrada de Ca2+ através dos canais de Ca2+ tipo L.

Apesar dos dados bem apresentados e experimentos devidamente realizados com conclusões bem fundamentadas, algumas questões devem ser analisadas em relação ao trabalho publicado. Em primeiro lugar, é importante destacar que, embora vários terpenos exibam ações cardiovasculares, incluindo propriedades antiarrítmicas, há muito pouca ou nenhuma evidência pré-clínica bem delineada de seu potencial para se traduzir na prática médica. Pode-se então argumentar se vale a pena estudar os terpenos para esse fim. Para adicionar mais dúvidas a este assunto, a maioria desses terpenos tem baixa potência farmacológica quando comparados a outros antiarrítmicos classe IV usados clinicamente como fenilalquilaminas,^12^ como o Verapamil. Mesmo quando as propriedades farmacológicas dos terpenos ocorrem na ordem de micromolar (cerca de 0,3 mM para a corrente de Ca2+, de acordo com os achados dos autores para (-)-Carvona), muitos terpenos têm múltiplos alvos que podem levar a vários efeitos colaterais indesejáveis. Os autores sugerem que o Qtc prolongado pode ser devido à possíveis efeitos da Carvona em outros canais iônicos. De fato, outras evidências suportam a capacidade da (-)-Carvona em modular outros canais, como canais de potencial receptor transitório (TRP).^13^

Com todas essas questões levantadas, qual é o verdadeiro potencial da (-)-Carvona e outros terpenos para o campo cardiovascular? Do meu ponto de vista, agora é a hora de explorar exatamente essas características de múltiplos alvos e potência relativamente baixa de (-)-Carvona e outros novos medicamentos à base de plantas com o objetivo de otimizar condições cardiovasculares específicas. A (-)-Carvona demonstrou experimentalmente ter efeitos antiparasitários, anticonvulsivantes, antidiabéticos, anti-inflamatórios, anticancerígenos e imunomoduladores, entre outros.^14^ Recentemente, a (-)-Carvona também demonstrou atenuar a toxicidade da doxorrubicina enquanto potencializa seus efeitos antitumorais.^15^ Portanto, a triagem das propriedades biológicas dos terpenos tem um vasto potencial para criar novas e otimizadas terapias para doenças cardiovasculares, especialmente em combinação com drogas já estabelecidas.

Para abordar essas questões de forma mais abrangente, estudos futuros devem se concentrar no uso de (-)-Carvona e outros terpenos em modelos específicos de doenças cardiovasculares, explorando suas propriedades biológicas atualmente investigadas. Além disso, ainda faltam na literatura informações sobre a farmacocinética e farmacodinâmica de muitos desses compostos e sua toxicidade após exposição aguda e prolongada. Em geral, a (-)-Carvona e outros terpenos têm potencial para serem traduzidos para a prática clínica, seja como droga antiarrítmica ou devido a outras de suas muitas ações biológicas; no entanto, estudos futuros são necessários, abrangendo condições cardiovasculares mais específicas e comparando as terapias atualmente utilizadas com essas novas abordagens usando (-)-Carvona e outros medicamentos derivados de plantas.
