# Supplementary material for: Para Onde Vamos com os Produtos Naturais? Explorando o Verdadeiro Potencial de Novos Medicamentos Derivados de Plantas no Campo Cardiovascular
Source: Arq Bras Cardiol. 2022 Jul 29;119(2):305–6. [Article in Portuguese] doi: 10.36660/abc.20220430 (PMC9363053; doi:10.36660/abc.20220430)
Supplement: Translation

# Translation: Where are We Going with Natural Products? Exploring the True Potential of New Plant-Based Drugs in the Cardiovascular Field

**Authors:** Artur Santos-Miranda

**Affiliations:** 1 Belo Horizonte MG Brazil Universidade Federal de Minas Gerais, Belo Horizonte, MG – Brazil

**Keywords:** Cardiovascular Diseases/physiopathology, Anti-Arrhythmics, Risk Factors, Cardiotoxicity, Medicinal Plants, Patch-Clamp Techniques, Terpenes, Carvone

Cardiovascular diseases are a leading cause of death worldwide. In the Brazilian population, it is estimated that approximately 41.6% of women and 63.5% of men are at medium to high risk of developing cardiovascular diseases in the next 10 years.^1^ Cardiac arrhythmias are common manifestations of cardiovascular diseases and configure an important cause of morbidity and mortality among cardiac diseases. After Vaughan-Williams’ classification of antiarrhythmic drugs based on their pharmacological actions, several new therapies and drugs were proposed, aiming to achieve a high efficacy with the least adverse effects. However, treatments with antiarrhythmic drugs and other agents used to treat cardiovascular conditions such as heart failure are often prone to pro-arrhythmic adverse responses.^2,3^ In addition, cardiac complications, such as arrhythmias, are also observed in treating other pathologies, including cancer, and during the use of antidepressants.^4,5^

Plant-based medicines have long been used in traditional/alternative medicine for the most diverse purposes. Its uses correlate with several factors, including family tradition, age, sex, education, socioeconomic status, and failure of conventional therapies.^6^ Among plant-based drugs, different types of terpenes have been explored as fragrances/repellents but also according to their medical potential in treating parasitic diseases, bacterial infection, wound healing, and as anti-inflammatory antioxidant agents.^7^ Moreover, the antiarrhythmic properties of some terpenes have been addressed using *in vitro* and experimental model approaches,^8,9^ while other terpenes might actually have pro-arrhythmogenic activity.^10^

In this issue of the *Arquivos Brasileiros de Cardiologia*, the antiarrhythmic properties of the monoterpene (-)-Carvone was explored *in vitro* and *ex vivo* using diverse preparations that range from cellular assays to the isolated organ.^11^ (-)-Carvone evoked a negative inotropic effect in the atria in a concentration-dependent fashion and reduced the contractility of isolated hearts after acute exposure to the terpene. The electrocardiogram (ECG) profile of isolated hearts exposed to this drug was marked with decreased heart rate, increased PR interval, and QTc. In freshly isolated cardiomyocytes, (-)-Carvone led to a decreased L-type calcium current, intracellular calcium transient, and cellular contraction, which aligns well with their isolated heart and atria findings. On top of their findings, (-)-Carvone reduced the severity of arrhythmias in an experimental model of isolated hearts exposed to a high Ca^2+^ media. The authors concluded that (-)-Carvone has a promising antiarrhythmic activity by decreasing Ca^2+^ entry through L-type Ca^2+^ channels.

Despite the well-presented data and properly performed experiments with well-supported conclusions, some questions are to be analyzed regarding the published work. First, it is important to highlight that although several terpenes display cardiovascular actions, including antiarrhythmic properties, there is very little, if any, well-conducted pre-clinical evidence of their potential to translate into the medical practice. One could then argue if terpenes are worth studying to this end. To add more doubt to this matter, most of these terpenes have low pharmacological potency when compared to other clinically used class IV antiarrhythmic like phenylalkylamines,^12^ like Verapamil. Even when terpenes’ pharmacological properties fall in the low micromolar range (around 0.3 mM for the Ca^2+^ current, according to the authors’ findings for (-)-Carvone), many terpenes have multiple targets that could predictably lead to several undesirable side effects. In fact, the authors suggest that the prolonged Qtc may result from (-)-Carvone off-targets on other ion channels. Indeed (-)-Carvone was shown to activate other channels such as transient receptor potential (TRP) channels.^13^

With all these issues raised, what is the true potential of (-)-Carvone and other terpenes to the cardiovascular field? From my point of view, the time is now to explore exactly these features of multi targets and relative low potency of (-)-Carvone and other plant-based new drugs aiming to optimize specific cardiovascular conditions. (-)-Carvone has been demonstrated experimentally to have antiparasitic, anti-convulsant, antidiabetic, anti-inflammatory, anti-cancer, and immunomodulatory effects, among others.^14^ Recently, (-)-Carvone was also shown to attenuate doxorubicin toxicity while potentiating its antitumoral effects.^15^ Therefore, screening the biological properties of terpenes has a vast potential to create new and optimized therapies for cardiovascular diseases, especially in combination with already established drugs.

To address these questions more comprehensively, future studies should be focused on using (-)-Carvone and other terpenes in specific models of cardiovascular diseases, exploring their biological properties that are currently investigated. Moreover, information on the pharmacokinetics and pharmacodynamics of many of these compounds and their toxicity after acute and long-term exposure are still lacking in the literature. Overall, (-)-Carvone and other terpenes do have a potential to be translated to clinical practice, either as an antiarrhythmic drug or due to other of its many biological actions; however, future studies are needed, covering more specific cardiovascular conditions and comparing currently used therapies with these new approaches using (-)-Carvone and other plant-based drugs
